# Trends in corrected lung cancer mortality rates in Brazil and regions

**DOI:** 10.1590/S1518-8787.2016050006209

**Published:** 2016-06-17

**Authors:** Deborah Carvalho Malta, Daisy Maria Xavier de Abreu, Lenildo de Moura, Gustavo C Lana, Gulnar Azevedo, Elisabeth França

**Affiliations:** I Departamento Materno-Infantil e de Saúde Pública. Escola de Enfermagem. Universidade Federal de Minas Gerais. Belo Horizonte, MG, Brasil; II Núcleo de Educação em Saúde Coletiva. Faculdade de Medicina. Universidade Federal de Minas Gerais. Belo Horizonte, MG, Brasil; III Organização Pan Americana de Saúde. Brasília, DF, Brasil; IV Programa de Pós-Graduação em Estatística. Instituto de Ciências Exatas. Universidade Federal de Minas Gerais. Belo Horizonte, MG, Brasil; V Instituto de Medicina Social. Universidade do Estado do Rio de Janeiro. Rio de Janeiro, RJ, Brasil; VI Programa de Pós-Graduação em Saúde Pública. Faculdade de Medicina. Universidade Federal de Minas Gerais. Belo Horizonte, MG, Brasil

**Keywords:** Lung Neoplasms, mortality, Mortality, trends, Underregistration, Mortality Registries

## Abstract

**OBJECTIVE:**

To describe the trend in cancer mortality rates in Brazil and regions before and after correction for underreporting of deaths and redistribution of ill-defined and nonspecific causes.

**METHODS:**

The study used data of deaths from lung cancer among the population aged from 30 to 69 years, notified to the Mortality Information System between 1996 and 2011, corrected for underreporting of deaths, non-registered sex and age , and causes with ill-defined or garbage codes according to sex, age, and region. Standardized rates were calculated by age for raw and corrected data. An analysis of time trend in lung cancer mortality was carried out using the regression model with autoregressive errors.

**RESULTS:**

Lung cancer in Brazil presented higher rates among men compared to women, and the South region showed the highest death risk in 1996 and 2011. Mortality showed a trend of reduction for males and increase for women.

**CONCLUSIONS:**

Lung cancer in Brazil presented different distribution patterns according to sex, with higher rates among men and a reduction in the mortality trend for men and increase for women.

## RESUMO

**OBJETIVO:**

Descrever a tendência da mortalidade por câncer de pulmão no Brasil e regiões, antes e após as correções por sub-registro de óbitos, redistribuição de causas mal definidas e causas inespecíficas.

**MÉTODOS:**

Foram utilizados dados de óbitos por câncer de pulmão da população de 30 a 69 anos, notificados ao Sistema de Informação sobre Mortalidade, entre 1996 e 2011, corrigidos para sub-registro de óbitos, declaração de sexo e idade ignorados e causas com códigos mal definidos e inespecíficos segundo sexo, idade e região. Foram calculadas taxas padronizadas por idade para dados brutos e corrigidos. Realizou-se análise da tendência temporal da mortalidade por câncer de pulmão por meio do modelo de regressão com erros autorregressivos.

**RESULTADOS:**

O câncer de pulmão no Brasil apresentou taxas mais elevadas em homens que em mulheres e a região Sul foi a que apresentou maior risco de morte em 1996 e 2011. A mortalidade tendeu a reduzir para o sexo masculino e a aumentar para o sexo feminino.

**CONCLUSÕES:**

O câncer de pulmão no Brasil apresenta padrão de distribuição diferente segundo sexo, com taxas mais elevadas em homens e com redução da tendência de mortalidade para o sexo masculino e aumento das taxas para o sexo feminino.

## INTRODUCTION

Cancer is a public health issue and ranks second in mortality around the world; in some developed countries, it has become the first leading cause of death^a^. Among neoplams, lung cancer has been the most common type worldwide for several decades^b^. According to the World Health Organization (WHO), in 2012 there were approximately 1.8 million new cases of lung cancer, or 13.0% of total cancer, of which 58.0% occurred in developing countries. It is estimated that growth rates grows 2.0% every year^b^.

The five-year survival rate for this disease ranges from 13.0% to 21.0% in developed countries, and from 7.0% to 10.0% in developing countries. Overall differences are greater in incidence, mortality and survival, and, in general, geographic patterns of mortality accompany those related to incidence^b^. In Brazil, lung cancer is the leading cause of death from cancer among men and the second among women. In 2011, it caused 22,424 deaths in all ages, 13,698 among men and 8,726 among women^c^.

The association between smoking and lung cancer was first suggested in England, in 1927^4^ ^,^ ^22^. Later studies showed that the interruption of smoking reduces the risk of lung cancer^d^. Therefore, studies show that incidence rates of lung cancer in a given country reflect the prevalence of smoking among its population^b^ ^,^ ^d^.

Although it still prevails as the leading cause of deaths by cancer worldwide^3^ ^,^ ^7^, lung cancer mortality rates have decreased. This decrease is heterogeneous and mortality from lung cancer in Eastern Europe, China and other developing countries is still increasing among both sexes^21^ ^,^ ^b^. In Brazil, in 1989, the *Pesquisa Nacional em Saúde e Nutrição* (National Health and Nutrition Survey) collected nationally representative data on smoking for the first time and found prevalence of 40.0% among men and 26.0% among women in the population over 15 years of age^16^. Since 2006, the Brazilian Noncommunicable Diseases Surveillance System (VIGITEL) has annually monitored the prevalence of smoking in the adult population in state capitals, and the results indicate decreasing trends among men and women, reaching in 2013 the average value of 11.3%, higher among men (14.4%) than women (8.6%). However, in some capitals in the South and Southeast regions, prevalence of smoking among women is already approaching the rates observed among men^13^ ^,^ ^e^.

At the World Health Assembly in 2014, WHO endorsed the Global Action Plan for the Prevention and Control of Noncommunicable Diseases^e^, which establishes a commitment among countries to reduce mortality in the four groups of noncommunicable diseases (NCD) to 25.0% by 2025, and to continuously monitor trends and improve data quality. Data improvement is especially relevant for Brazil, since, despite the consolidation of the Mortality Information System (SIM), there is still underreporting of deaths and a high proportion of deaths recorded with ill-defined causes in some regions of the country. Therefore, analysis of the magnitude of mortality rates must consider the underreporting of deaths and the occurrence of ill-defined or garbage codes, as they introduce a bias in comparisons between areas with differences in quality of information on deaths and in time trend studies.

Considering the importance of lung cancer in the epidemiological profile of the Brazilian population^18^ and the persisting problems regarding the quality of death reports in the country, mortality analyses should incorporate methods to correct underreporting of deaths and redistribute ill-defined and nonspecific causes of death. Following such adjustments, time analyses aimed at determining the existence of significant increasing or decreasing trends are important to assess whether mortality rate reduction targets are being met. Different results over the years may be merely the result of random fluctuation, and not of actual improvements or setbacks.

The aim of this study was to describe the trends in lung cancer mortality in Brazil and regions before and after correction for underreporting of deaths and redistribution of ill-defined and nonspecific causes.

## METHODS

This study is a time series analysis of mortality based on deaths reported to SIM between 1996 and 2011, available online from the Brazilian Unified Health System Information Department (DATASUS)^b^. The age group from 30 to 69 years was selected for the study, due to the priority given to monitoring deaths considered premature, as recommended by WHO in the Global Action Plan for the Prevention and Control of NCD^e^ and the strategic action plan for the prevention and control of noncommunicable diseases in Brazil^12^ ^,^ ^f^.

The codes of the International Classification of Disease (ICD-10) considered for trachea, bronchus and lung cancer were: C33-C34.9, D02.1-D02.2, D38.1. Raw data of deaths reported to SIM were corrected according to the following methodological procedures:

- Step 1: imputation of deaths with non-registered sex or age;
- Step 2: redistribution of some nonspecific causes allocated in the chapters of defined causes of ICD-10 (all chapters except Chapter XVIII), here called “garbage” codes, by sex and region;
- Step 3: redistribution of death causes allocated in Chapter XVIII of ICD-10 of ill-defined causes (IDC), by sex, age group and region;
- Step 4: correction of underreporting of deaths, with computation of total deaths corrected for underreporting in each five-year age group of 30 to 69 years by sex, and allocation of the proportion of the specific causes corrected in the previous steps.

The specific rates by cause were calculated in the five-year age groups by sex and region for the data corrected in the described steps above. To analyze the rates of the total 30-69 age group, standardization was carried out using the direct method of specific rates by cause (corrected) of the age groups for Brazil and the different regions, by sex. These rates were then used for the analysis of time trends from 1996 to 2011.

Following the evaluation of the list of causes and the garbage code list of the Global Burden of Disease 2010 Study (GBD-2010)^10^ ^,^ ^17^, specifics ICD-10 codes were defined to be included in each grouping of garbage codes, with redistribution criteria for the target diseases or target codes. Table 1 details the ICD-10 codes that were included in each group of garbage codes.

**Table 1:** List of garbage codes and redistribution percentage for lung cancer according to the Global Burden of Disease 2010 (GBD-2010) Study.

Garbage codes^a^	Garbage codes (ICD-10 codes)^b^	Redistribution (%)
Disseminated intravascular coagulation, acute respiratory failure, cardiac arrest	D65, I46, J96.0, J96.9	3.31
Cerebral palsy and other paralytic syndromes	G80-G83	0.79
Embolism and thrombosis	I74, I81	0.56
Ill-defined of A00-B99	A59-A60.0, A60.9, A63-A64, A71-A74, B07-B09, B35-B36, B74.4-B74.8, B75, B85-B88, B95-B97	2.89
Ill-defined of F30 F99	F30-F33, F34.1, F40-F48, F51-F53, F60-F99	4.80
Ill-defined of G43-G58.9	G43-G44, G47-G52, G54, G56-G58	4.89
Ill-defined of H00-H99	H00-H02, H04-H05, H10-H11, H15-H18, H20-H21, H25-H26, H30-H31, H33-H35, H43-H47, H49-H57, H60-H61, H69, H71-H74, H80-H81, H83-H93	2.69
Ill-defined of J30-J35.9	J30, J33, J34.2, J35	2.31
Ill-defined of K00-K14.9	K00-K11, K14	3.61
Ill-defined of L01-L98.9	L3.1, L4, L20-L30, L45, L50, L52-L68, L70-L85, L90-L92, L98	4.13
Ill-defined of M09-M99	M10-M11, M15-M25, M40, M45, M47-M48, M50-M60, M65-M67, M70-M71, M75-M79, M95-M99	4.42
Ill-defined of N39.3-N97.8	N39.3, N40, N45-N46, N60, N84-N92, N95, N97	4.46
J81 Pulmonary edema	J81	0.04
N17-N19 Renal insufficiency	N17-N19	0.01
Non-specified liver diseases	K71.0-K71.6, K71.8-K72.0, K75.0-K75.1	0.21
Ill-defined of D10-D36.9	D10-D24, D26-D31, D35-D36	4.86
Ill-defined of Q10-Q84.9	Q16-Q18, Q36, Q54, Q65, Q67-Q68, Q72-Q74, Q82-Q83	1.53
Pleural and chest diseases	J86-J90, J93.8-J94	0.67
Neoplasm of uncertain or unknown behavior not otherwise specified	D00.0, D01.7-D01.9, D02.3-D02.9, D07.3, D07.6, D09.7-D09.9, D37.9, D38.6, D39.9, D40.9, D41.9, D48.9	5.52
C39.0-9, Malignant neoplasm of other sites and of ill-defined sites in the respiratory system and intrathoracic organs	C39	80.83
C76.0-9, Malignant neoplasm of other sites and of ill-defined sites	C76	3.86
C80.0-9, Malignant neoplasm with no specific site	C80	2.57

^a^ Adapted from Lozano et al.^10^ (2012)

^b^ Defined by this study.

In this study, however, IDC (ICD-10 R codes) were redistributed according to the methodology proposed by França et al.^5^, which considers the availability of information on death certificate investigationsof IDC carried out by Brazilian state and municipal health departments since 2006. The procedure of redistributing IDC according to investigation results was used for the period 2006-2011, based on percentages of NCD found among the reclassified IDC from each year. Given that these investigations were not initiated before 2006, the average redistribution values for the period 2006-2011 were used for the period 1996-2005.

Correction of underreporting of deaths in Brazil and regions was carried out using the estimates provided by the Health Interagency Information Network (RIPSA) for Brazil and regions of total deaths for the period 1996-2011, for both sexes^g^. For the period 1996-1999, RIPSA used the underreporting correction factors based on the ratio between the number of deaths reported to SIM and the number of deaths estimated by IBGE. Between 2000 and 2011, the correction factors were based on the number of deaths estimated by an active search for unreported deaths and births^20^. Although the factors estimated through active searching are considered more reliable, this method had only been applied as of 2000. Therefore, for the period between 1996 and 1999, the correction factors provided by RIPSA^f^ were used. As RIPSA^f^ has no coverage by sex, the mean estimates presented by Agostinho and Queiroz^h^ were used.

For the standardization of mortality rates, the study used as the standard population the Brazilian population of 2010, since this is the most recent Census^i^, being close to the age distribution of the current population, which is still in the process of demographic transition.

Following the corrections, the study used the regression model for the time series analyses of each region and Brazil by sex^1^. The trend was defined as *µ*_*t*_ *= ß*_*0*_ *+ ß*_*1*_ *t*, implying a growth (if the *ß*_*1*_ slope is positive) or decrease (if the slope is negative) in a straight line over time. The *ß*_*1*_ slope informs by how many units the expected value of this series increases or decreases each year. Moreover, since the data indicated a sign of autocorrelation in the first order, it was assumed that the model’s residuals (the difference between *µ*_*t*_ and the observed value) follow the autoregressive model of order one. Hypothesis testing defines whether the slope value is significantly different from zero or not. The regression model has the function of clearly indicating the nature of the trend and whether it is significant or not.

This study was prepared with aggregate secondary data of deaths and populations, obtained from the Brazilian Ministry of Health databases published on the Internet. The consulted databases did not include sensitive information such as names and addresses, therefore approval by an ethics research committee was not required for the study.

## RESULTS

Among the steps of correction and redistribution of garbage codes, IDC and underreporting of deaths, the latter was the largest contributor to the total number of deaths for both men and women in the two years analyzed. At the end of all correction steps, the number of deaths among men increased by 20.4% in 1996 and 10.1% in 2011. The same was observed for women, ranging from 34.2% in 1996 to 10.0% in 2011 (Table 2).

**Table 2:** Participation of each redistribution step in the total number of deaths for lung cancer, according to sex. Brazil, 1996 and 2011.

Men					Women				
	
1996									
		1^st^ position (%)	2^nd^ position (%)	3^rd^ position (%)			1^st^ position (%)	2^nd^ position (%)	3^rd^ position (%)
Absolute number before correction	Absolute number after correction	Under-reporting	Ill-defined causes	Garbage codes	Absolute number before correction	Absolute number after correction	Under-reporting	Ill-defined causes	Garbage codes
5,722	6,943	12.0	5.8	2.6	21,353	2,951	17.9	11.0	5.3

2011									

		1^st^ position (%)	2^nd^ position (%)	3^rd^ position (%)			1^st^ position (%)	2^nd^ position (%)	3^rd^ position (%)
Absolute number before correction	Absolute number after correction	Under-reporting	Garbage codes	Ill-defined causes	Absolute number before correction	Absolute number after correction	Under-reporting	Garbage codes	Ill-defined causes
7,390	8,157	4.8	2.8	2.5	5,020	5,536	5.5	2.8	1.7

Deaths and standardized death rates from lung cancer showed significant variation following correction and redistribution of deaths for men and women in the North and Northeast regions in 1996. This effect was smaller in 2011 for both sexes in these and the other regions (Table 3).

**Table 3:** Lung cancer deaths and standardized mortality rates* among ages 30 to 69 by region. Brazil, 1996-2011.

Region	1996				Rate % variation	2011				Rate % variation
	
	Initial		Final			Initial		Final		
			
	n	rate	n	rate		n	rate	n	rate	
Men										
North	198	14.4	397	28.5	97.9	310	12.0	400	15.4	28.3
Northeast	553	8.6	1,261	19.5	126.7	1,204	11.9	1,461	14.4	21.0
Southeast	1,518	36.6	1590	38.3	4.6	1,966	29.3	2,072	30.8	5.1
South	3,154	27.0	3,329	28.5	5.6	3,426	18.3	3,691	19.7	7.7
Midwest	299	19.6	366	23.9	21.9	484	17.2	533	18.9	9.9
Brazil	5,722	22.7	6,943	27.5	21.1	7,390	18.0	8,157	19.9	10.6
Women										
North	74	5.5	164	12.2	121.8	206	8.1	250	9.8	21.0
Northeast	270	3.7	763	10.3	178.4	986	8.5	1,185	10.2	20.0
Southeast	569	12.5	620	13.6	8.8	1,170	15.7	1,230	16.5	5.1
South	1,116	8.3	1,240	9.3	12.0	2,338	10.8	2,519	11.7	8.3
Midwest	123	8.0	164	10.6	32.5	320	10.5	351	11.5	9.5
Brazil	2,152	7.6	2,951	10.4	36.8	5,020	10.9	5,535	12.0	10.1

* Standardized by age according to the Brazilian population in 2010.

Following the correction and redistribution of deaths, death rates from lung cancer among the population aged 30-69 years were higher for men in 1996 and 2011. The South region had the highest rate for men and women. The lowest rates were observed for men in the Northeast region in the two years analyzed and for women in the Southeast (1996) and North (2011) regions. For women, there was increased risk in the Southeast, South and Midwest regions and in the country as a whole. This also caused a convergence trend of mortality rates between regions for women (Table 3).

Mortality rates for lung cancer increase in risk according to age. This was observed for men and women in 1996 and 2011. Men have higher mortality rates than women, particularly over 55 years of age. This phenomenon is common to all regions and the country as a whole. However, the difference decreased in 2011 (Table 4).

**Table 4:** Lung cancer deaths and standardized mortality rates before and after correction according to age and sex. Brazil, 1996 and 2011.

Age	1996				2011			
	
	Initial		Final		Initial		Final	
			
	n	rate	n	rate	n	rate	n	rate
Men								
30-34	46	0.8	87	1.5	44	0.6	66	0.8
35-40	103	1.9	159	2.9	86	1.3	114	1.7
40-44	249	5.5	332	7.3	201	3.1	246	3.8
45-49	450	12.3	576	15.8	436	7.5	505	8.6
50-54	684	22.9	834	27.9	982	19.6	1,090	21.7
55-59	1,057	46.3	1,267	55.5	1,551	37.8	1,685	41.1
60-64	1,501	77.9	1,752	91.0	2,023	63.9	2,200	69.5
65-69	1,631	110.7	1,936	131.4	2,067	89.9	2,250	97.8
Total	5,721	22.7	6,943	27.5	7,390	18.0	8,156	19.9
Women								
30-34	39	0.6	70	1.1	48	0.6	61	0.8
35-40	77	1.4	125	2.2	88	1.2	106	1.5
40-44	138	2.9	200	4.2	221	3.3	250	3.7
45-49	225	5.8	308	8.0	448	7.1	500	7.9
50-54	289	9.2	391	12.4	821	14.9	898	16.3
55-59	367	14.6	495	19.7	1,087	23.6	1,187	25.8
60-64	476	21.8	638	29.3	1,099	30.5	1,208	33.5
65-69	542	31.6	725	42.2	1,208	44.8	1,326	49.2
Total	2,153	7.6	2,952	10.4	5,020	10.9	5,536	12.0

For uncorrected data, all regions showed a downward trend for men, significant or not, except for the Northeast region, which showed a significant increasing trend. After correction, all regions showed a significant downward trend for men (Figure).

**Figure:**
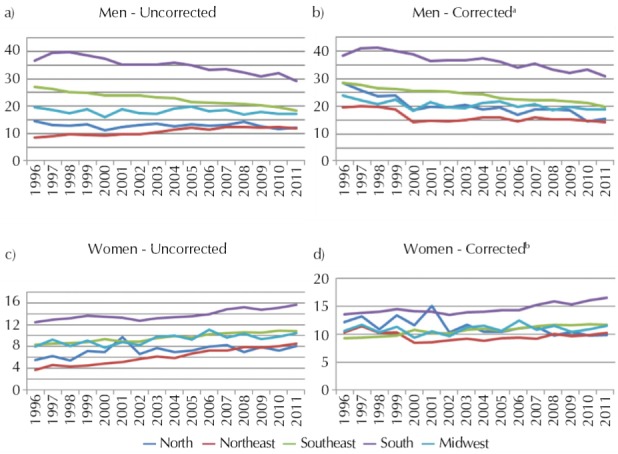
Lung cancer mortality trends according to sex and geographic regions. Brazil, 1996-2011.

^a^ Slope and p (in parentheses) value for corrected data: North: -0.706 (0.000), Northeast: -0.325 (0.016), Southeast: -0.531 (0.000), South: -0.607 (0.000), Midwest: -0.204 (0.002).

^b^ Slope and p (in parentheses) value for corrected data: North: -0.213 (0.000), Northeast: -0.022 (0.726), Southeast: 0.175 (0.000), South: 0.177 (0.002), Midwest: 0.041 (0.207).

For women, all regions showed a significant increase for uncorrected data (Figure). After correction, the South and Southeast regions maintain the same pattern, but the North region has shown a significant downward trend.

## DISCUSSION

Distribution patterns of lung cancer in Brazil differ according to sex, with higher rates among men than women, and reduced mortality trends for men and increased rates for women.

The difference in the occurrence of lung cancer according to sex is related to the distinct characteristics of exposure to smoking among men and women^3^. The analysis of the variation in mortality trends according to sex and geographical distribution is based on the different behaviors in tobacco use by sex over time.

Studies show increased mortality from lung cancer since the 1940s. In Europe, mortality has stabilized or decreased among men and increased among women in the last decades^2^ ^,^ ^8^ ^,^ ^15^. Most studies attribute this variation to changes in smoking habits, with trends declining among men and rising among women in most countries^2^ ^,^ ^6^ ^,^ ^8^ ^,^ ^15^.

Differences in smoking habits in the past have shown a much lower risk of lung cancer among women^22^. Women used to start smoking later and smoked fewer cigarettes per day. As the prevalence of smoking among women increased in many countries, mortality by sex tends to equalize^2^ ^,^ ^6^ ^,^ ^8^ ^,^ ^15^.

Malta et al.^11^ analyzed the trend in mortality from lung cancer between 1980 and 2003 for Brazil and selected states. The authors observed a reduction among men up to 59 years of age and an increase among women of all age groups from 30 years on. The maintenance of high mortality rates in older populations is due to smoking habits prevalent in the past^9^.

Silva et al.^18^, analyzing mortality from lung cancer in Brazil over the period 1979-2003, identified an increase of 29.0% among men and 86.0% among women. Analysis by region showed that in the Southeast region, between 1996 and 2003, the youngest age group (40-59 years) showed a decrease in mortality among men and an increase among women. Analysis by age showed the same pattern for this age group.

Therefore, the results of this study follow the same trend observed in Brazil and other countries, with declining mortality among men and an increasing trend among women^2^ ^,^ ^6^ ^,^ ^8^. Despite the recent decline , the higher rates observed among men reflect past habits, i.e., a higher prevalence of smoking among the male population, whose smoking habit precedes that of women^22^. In Brazil, women began using tobacco later, with smoking promoted as a desired behavior in movies and the media, alongside the strong cultural appeal of assertion of independence and women’s liberation^13^. Studies indicate that the peak of tobacco consumption will impact mortality rates approximately 30 years later^9^ ^,^ ^19^. Besides smoking, genetic, hormonal and physiological factors, and also interaction between them, may have a specific impact on the lung carcinogenesis process among women^3^.

The attributable risk of smoking as a causative agent of lung cancer is higher than 90.0%^22^. Other factors also participate in the etiology of this disease^22^, but with substantially lower attributable risk, ranging from 1.0% to 10.0% with environmental pollution. For example, radon gas has an attributable risk of about 1.0%^22^; asbestos and other mineral fibers, about 4.0%; chromium, nickel and arsenic, about 4.0%; and exposure to silica, 1.5%^22^.

Brazil has a strong tradition in confronting smoking. With the 2011-2022 Strategic Action Plan the Prevention and Control of NCD^12^, the country committed itself to reducing mortality from NCD, among them cancer, and reducing smoking by 30.0% within a decade. These goals have been achieved thanks to regulatory, educational and health promotion measures implemented in the last decade^12^. In 2005, Brazil joined the Framework Convention on Tobacco Control. In 2011, tobacco legislation was improved with Law 12,546/2011. In 2014, Presidential Decree 8,262/2014 on smoke-free environments was approved, banning indoor smoking, as well as all cigarette advertising. The Decree additionally expanded the space occupied by health warnings on cigarette packs by 30.0% and increased tobacco taxation, besides setting a minimum price for cigarettes^14^.

The trends observed in this study (population from 30 to 69 years), however, may not occur among the older population. In addition, correction of death underreporting considered the whole population aged five and over, including the elderly, who are more prone to death undercounting. This strategy may overestimate mortality, since death records of the younger population tend to be more precise compared to the older population.

Mortality analyses using methods to redistribute garbage codes and correct underreporting of deaths reduce problems of underestimation of mortality rates by specific cause, in addition to being more appropriate for comparative analyses between regions and time trend analysis. The contribution of each stage in the process of correcting raw death data enables the establishment of strategies to improve information sources and build analysis proposals. In addition, mortality indicators with better quality of information can be more adequately used in public administration.

Lung cancer has a long latency period; therefore, the reduced trend in early mortality rates among men identified in this study results from national efforts to reduce the prevalence of smoking in the country in recent decades. These results indicate the importance of public health measures in protecting life and in reducing preventable deaths.

## Footnotes

a World Health Organization. Global status report on noncommunicable diseases 2010 [Internet]. Geneva: World Health Organization; 2011 p. 176.

b International Agency for Research on Cancer. Lung Cancer: estimated incidence, mortality and prevalence worldwide in 2012. Lyon: International Agency for Research on Cancer; 2012 [cited 14 Nov 2014]. Available from: http://globocan.iarc.fr/Pages/fact_sheets_cancer.aspx?cancer=lung

c Ministério da Saúde. SIM - Sistema de Informação sobre Mortalidade. Brasília (DF); 2008 [cited 14 Nov 2014]. Available from: http://www2.datasus.gov.br/DATASUS/index.php?area=060701

d World Health Organization. WHO report on the global tobacco epidemic, 2011: warning about the dangers of tobacco. Geneva: World Health Organization; 2011 [cited 14 Nov 2014]. Available from: http://www.who.int/tobacco/global_report/2011/en

e World Health Organization. WHO Global NCD Action Plan 2013-2020. Geneva: World Health Organization; 2013 [cited 14 Nov 2014]. Available from: http://www.who.int/nmh/events/ncd_action_plan/en/

f Ministério da Saúde. Secretaria de Vigilância em Saúde. Departamento de Análise de Situação de Saúde. Plano de ações estratégicas para o enfrentamento das Doenças Crônicas Não Transmissíveis (DCNT) no Brasil 2011-2022. Brasília (DF): Ministério da Saúde; 2011 [cited 2012 Jun 17]. Available from: http://portal.saude.gov.br/portal/arquivos/pdf/cartilha_dcnt_pequena_portugues_espanhol.pdf

g Ministério da Saúde. Ficha de qualificação da RIPSA-2012. Demográficos. Razão entre óbitos informados e estimados - A.18 – 2012. Brasília (DF); 2012 [cited 2014 Aug 4]. Available from: http://fichas.ripsa.org.br/2012/a-18/?l=pt_BR

h Agostinho CS. Estudo sobre mortalidade adulta, para o Brasil entre 1980 e 2000 e unidades da Federação em 2000: uma aplicação dos métodos de distribuição de mortes [these]. Belo Horizonte (MG): Universidade Federal de Minas Gerais; 2009.

i Ministério da Saúde. DATASUS. População residente – Brasil. 2010 [cited 2014 Aug 4]. Available from: http://tabnet.datasus.gov.br/cgi/tabcgi.exe?ibge/cnv/popuf.def
